# Preliminary Study of Novel Bio-Crypto Key Generation Using Clustering-Based Binarization of ECG Features

**DOI:** 10.3390/s24051556

**Published:** 2024-02-28

**Authors:** Ho Bin Hwang, Jeyeon Lee, Hyeokchan Kwon, Byungho Chung, Jongshill Lee, In Young Kim

**Affiliations:** 1 Department of Biomedical Engineering, Hanyang University, Seoul 04763, Republic of Korea; hobin0215@hanyang.ac.kr (H.B.H.); jeyeonlee@hanyang.ac.kr (J.L.); 2 Information Security Research Division, Electronics and Telecommunications Research Institute (ETRI), Daejeon 34129, Republic of Korea; hckwon@etri.re.kr (H.K.); cbh@etri.re.kr (B.C.)

**Keywords:** biometrics, ECG, signal processing, bio-crypto key, authentication, cryptography, biomedical engineering

## Abstract

In modern society, the popularity of wearable devices has highlighted the need for data security. Bio-crypto keys (bio-keys), especially in the context of wearable devices, are gaining attention as a next-generation security method. Despite the theoretical advantages of bio-keys, implementing such systems poses practical challenges due to their need for flexibility and convenience. Electrocardiograms (ECGs) have emerged as a potential solution to these issues but face hurdles due to intra-individual variability. This study aims to evaluate the possibility of a stable, flexible, and convenient-to-use bio-key using ECGs. We propose an approach that minimizes biosignal variability using normalization, clustering-based binarization, and the fuzzy extractor, enabling the generation of personalized seeds and offering ease of use. The proposed method achieved a maximum entropy of 0.99 and an authentication accuracy of 95%. This study evaluated various parameter combinations for generating effective bio-keys for personal authentication and proposed the optimal combination. Our research holds potential for security technologies applicable to wearable devices and healthcare systems.

## 1. Introduction

With the annual surge in demand for wearable devices, safeguarding personal information has become increasingly crucial [1]. The cornerstone of robust security is in ensuring that only authorized organizations or individuals can access sensitive data [2]. Previous studies have proposed various approaches to address these security issues, including biometric authentication and encryption using physiological signals [3,4,5,6].

Biometrics, a method of individual identification, utilizes unique physiological features or behavioral patterns [7]. Common biometric indicators include facial recognition, iris patterns, fingerprints, and vein structures. However, the major concern with biometrics is the potential exposure and theft of biometric traces after use [8,9,10]. In response, recent researchers have focused on encrypting data using physiological signals or generating encryption keys [5,6,11,12]. Among these, the electrocardiogram (ECG) is notable for its invisibility, inimitability, comfort, and suitability, with researchers presenting the ECG as a promising security resource [13].

This study focuses on an encryption key approach that avoids storing or transmitting original biometric data, reducing the risks of personal information infringement. This approach involves generating an encryption key from a critical initial value known as the ‘seed’ [14]. Encryption keys, which are crucial for protecting sensitive information, must demonstrate randomness, secrecy, and high entropy. Therefore, establishing a stable and secure seed is essential for generating robust encryption keys.

However, ECG variability presents challenges in bio-crypto key (bio-key) research. While ECG properties enhance encryption key randomness, variability can negatively affect key stability. Key stability is the consistent reproduction of the generated key, while key randomness pertains to unpredictability. These issues have led to the proposal of novel approaches for key generation to achieve high stability, randomness, and personal authentication.

Several studies focus on generating encryption keys using physiological signals. Moosavi et al. [5] introduced a method based on two photoplethysmograms, meeting five NIST (National Institute of Standards and Technology) benchmarks, with an average Hamming distance of 47.56% between keys from distinct subjects. Zhang et al. [6] presented a key generation framework based on the inter-pulse interval (IPI), achieving a Hamming distance of 51.6% between subjects and passing 15 NIST benchmarks. Cho et al. [11] proposed generating seeds using an IPI-based bloom filter, obtaining a 10% false-positive error rate with a 2048-bit key. On the other hand, few studies have considered utilizing ECG features for encryption keys. Karimian et al. [13] introduced an approach derived from ECG features using the interval-optimized mapping bit allocation technique and wavelet-based features, demonstrating high reliability and entropy. Moosavi et al. [15] explored ECG feature-based key generation, determining the optimal number of bits for each ECG feature and subsequently generating encryption keys, achieving a Hamming distance of 48.13% between subject keys and adhering to five NIST benchmarks with a pass rate exceeding 0.8.

Despite these advancements, significant limitations exist in the current studies. First, reproducing an encryption key that corresponds to the registered key can be difficult due to the variability of physiological signals. Addressing this variability could allow continuous personal authentication. Second, security flexibility might be limited. Employing uniform seed lengths could allow attackers to predict or identify encryption patterns more easily. Finally, most studies struggle to validate the performance of personal authentication. Consequently, a test to determine whether individuals can be accurately distinguished using their encryption keys is crucial.

From this point of view, we aim to evaluate the possibility of a bio-key for personal authentication that combines stability, flexibility, and user convenience. This study makes three important contributions. First, we introduce an innovative approach that generates stable encryption keys considering ECG variability. This addresses factors such as an individual’s heart rate, psychological state, and shifts in measurement electrode location through methods like ECG normalization, clustering-based binarization, and a fuzzy extractor. Second, we propose a novel Dynamic Quantization method based on clustering, offering each user flexible seed generation and personalized security levels. To our knowledge, this is the first approach used to generate personalized seeds. Third, we designed a method capable of generating personal encryption keys using ECGs within a short time, offering substantial advantages for real-world applications and services. Finally, our work establishes a foundational framework for research on encryption key-based personal authentication. The methodology we propose has the potential to be expanded to other biometric systems and can act as a paradigm for future research.

The rest of the paper is structured as follows. Section 2 outlines the proposed method. In Section 3, we present the analysis results of the proposed method. Section 4 provides the discussion of the proposed method, and Section 5 concludes the paper and presents future plans.

## 2. Materials and Methods

**Figure 1:**
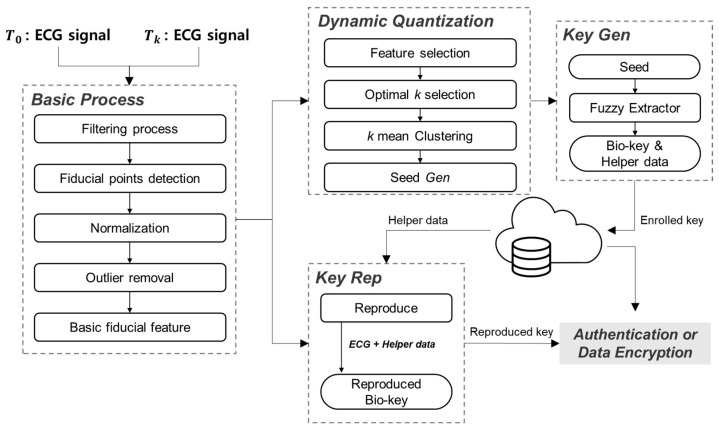
Overview of the proposed method and illustration of practical applications.

This study proposes a personalized bio-key generation method, as shown in Figure 1. We use specific data and basic processing, as described in Section 2.1 and Section 2.2, respectively. Section 2.3 explains the creation of the clustering model for Dynamic Quantization, while Section 2.4 outlines the bio-key generation process and the process of registration and authentication steps for the bio-key.

### 2.1. Dataset

The dataset consists of two parts. The ECG-ID dataset, sourced from PhysioNet, is utilized for building the clustering model described in Section 2.3 [16]. The experimental dataset is collected through experiments and serves as the primary data source for bio-key generation.

#### 2.1.1. ECG-ID Dataset

The ECG-ID database involves ECG data from 44 men and 46 women aged 13 to 75 years. The dataset consists of ECG lead I recordings captured for 20 s, sampled at 500 Hz and 12-bit resolution, with a nominal range of ±10 mV. The database contains at least two 20 s data segments from 90 subjects, and each file holds two distinct ECG data signals [17]. We used Signal 0, the unprocessed raw ECG lead I signal.

#### 2.1.2. Experimental Dataset

The experimental dataset for this study was derived from lead I ECG recordings. These recordings were obtained using a Biopac MP150 system (Biopac Systems Inc., Goleta, CA, USA) equipped with AcqKnowledge 4.2 software and an ECG100C amplifier. The ECG signals were sampled at a rate of 500 Hz with a measurement gain set to 1000. The dataset consisted of 10 healthy adults, seven males and three females, with no history of heart disease. The mean age of participants was 27.7 years, with a standard deviation of 2.31 years.

Data collection was conducted using dry electrodes, with each subject undergoing at least four measurement sessions at various times of the day. Subjects underwent ECG measurements by sitting at a table, wearing dry electrodes, and resting while ECG measurements were conducted for 20 s. Upon completion of the measurement, subjects removed the dry electrodes from their wrists and resumed their daily activities. Subsequently, subjects reseated themselves at the table at designated times, repositioned the dry electrodes on their wrists, and underwent another 20 s measurement. These repeated measurements were conducted at every time when subjects were available for measurement. Ethical approval for this research was granted by the Institutional Review Board (IRB) of Hanyang University (Approval HYI-16-030-1), and informed consent was obtained from all subjects before their participation.

### 2.2. Basic Process

#### 2.2.1. Noise Removal and Fiducial Point Detection

**Figure 2:**
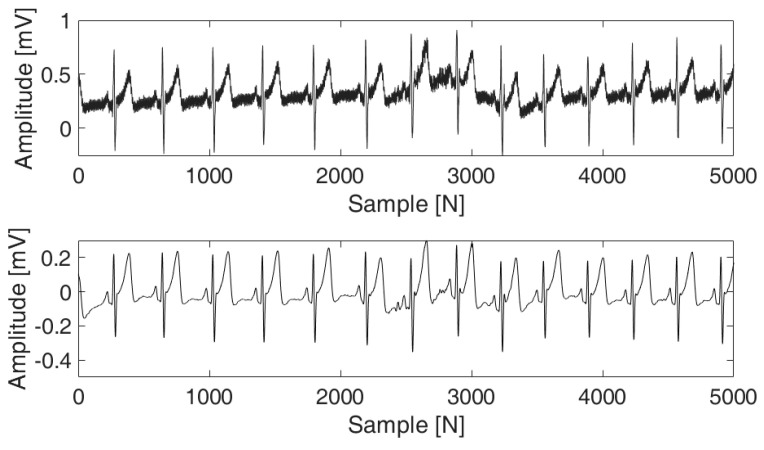
Graphs before and after noise removal.

ECG data acquired through dry electrodes are susceptible to raw signal contamination, such as baseline drift, power line interference, motion artifacts, and electromyography (EMG). These interferences introduce distortions into the original ECG signal; using such data to create a bio-key results in performance degradation. Thus, it is imperative to conduct noise reduction procedures. As shown in Figure 2, our approach involved application of a notch filter to effectively suppress power line interference at 60 Hz, complemented by a third-order Butterworth high-pass filter with a cutoff frequency of 0.5 Hz to eliminate baseline wander.

**Figure 3:**
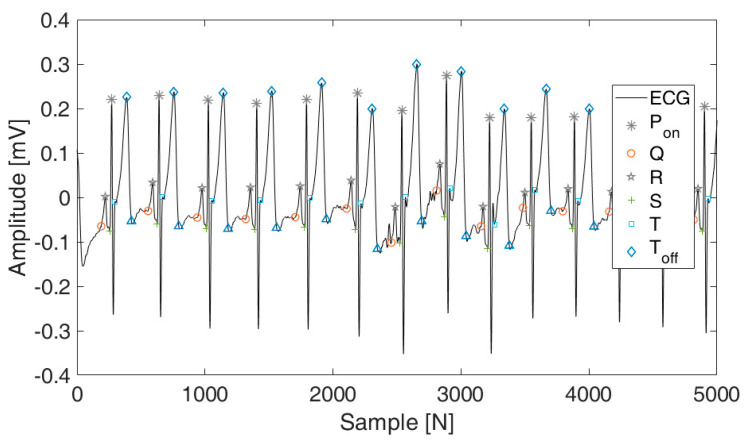
Continuous ECG-based fiducial point visualization graph.

We utilized a wavelet transform-based technique to identify reference points on the ECG after noise removal [18]. This method dissects the ECG signal into various scales, pinpointing key features including modulus maxima, minima, P wave onset (Pon), onset of QRS complex (QRSon), R peak (Rpeak), offset of QRS complex (QRSoff), and T wave offset (Toff), alongside zero-crossing points. The results of this algorithm are illustrated in Figure 3.

#### 2.2.2. Normalization

Non-linear normalization centers on adjusting ECG waveforms according to heart rate fluctuations to maintain the integrity of the authentication process [4]. It has been observed that heart rate changes led to non-linear alterations in the ECG signal. A previous study analyzed the ECG segment in four sections (PR interval, QRS complex, ST interval, and TP segment), and regression was performed against heart rate for each section [4]. Moreover, previous studies have validated that non-linear normalization enhances personal authentication performance at data collected under conditions inducing diverse heart rates.

We implemented non-linear normalization of each section to match the standard duration at 70 bpm. By integrating this nonlinear normalization approach into our methodology, we considered for the potential influence of ECGs obtained under various physiological conditions on the stability and reproducibility of the generated keys.

#### 2.2.3. Heartbeat Outlier Detection and Removal

Even when continuous ECG signals are normalized within each segment, waveforms deviating from the frequently occurring heartbeat waveform can introduce errors in bio-key generation. Consequently, we incorporated a step to eliminate outlier heartbeats from each ECG record file [19]. The procedure is outlined as follows:

1. We generated an ECG template (${ECG}_{t}$) using the entire ECG beat (${ECG}_{all}$) extracted from the ECG record through Equation (1).
  (1) $${ECG}_{t}={mean(ECG}_{all}),\;\;{ECG}_{all}=\left\{b\left(i\right)\right|\;1\leq i\leq N\},$$
  where b is the ECG beat, and N is the total count of ECG beats within the ECG record.
2. Outliers (${ECG}_{o}$) were detected using Equation (2).
  (2) $${ECG}_{o}=Find(\rho \left({ECG}_{all},\;{ECG}_{t}\right)<\mu_{\rho }-0.5\times \sigma_{\rho })$$
  where ρ is the correlation coefficient, $\mu_{\rho }$ is the mean of ρ, and $\sigma_{\rho }$ is the standard deviation of ρ.
3. Following removal of the detected ECG beats, we selected three ECG beats and applied Equation (3). Subsequently, the resulting ensemble beat (${ECG}_{\gamma }$) was used in the feature extraction process.
  (3) $${ECG}_{\gamma }=mean\left(f\left({ECG}_{all}-{ECG}_{o},\;3\right)\right)$$
  where f(k,l) is the function that selects the three ECG beats. Data are input into ‘k’ and the number of beats to be selected in ‘l’ is chosen.

#### 2.2.4. Widely Employed Feature Detection

Typically, ECG research focuses on two primary categories: fiducial and non-fiducial features. Among these, “fiducial features” are generally considered more stable and distinctive. These features are extracted based on specific points and exhibit lower sensitivity to errors or deformations, providing a consistent foundation for bio-key generation [19]. Moreover, this approach is simpler than non-fiducial methods and can result in rapid and efficient key generation for real-time authentication systems.

For this reason, we combined previously extracted P waves, QRS complexes, and T waves to extract 29 features [19]. These features can be categorized into four groups: amplitude features (features 1 to 9), duration features (features 10 to 18), slope and distance features (features 19 to 28), and area features (feature 29). The amplitude features measure the voltage difference between fiducial points, and duration features measure the time difference between fiducial points. The slope and distance features measure the change in voltage over time, and the area feature measures the area under the curve between fiducial points. Table 1 illustrates the extracted features from the ECG beat, including relative amplitude, temporal duration, slope, distance, and area, through fiducial points. We define the extracted 29 features as “feature set (*X*)” and as Equation (4):

(4) $$X=\left\{x(i)\right|\;1\leq i\leq 29\}$$

where x is the single feature extracted from the heartbeat.

### 2.3. Personalized Dynamic Quantization

#### 2.3.1. Proposed Scheme

This study introduces a clustering model designed for Personalized Dynamic Quantization. We aim to generate personalized seeds by selecting unique ECG features for each individual. Then, we examine multicollinearity by pairing ECG features and analyzing their interactions. Only the feature pairs that exhibit no multicollinearity are included in the clustering model. Subsequently, we determine the optimal number of clusters for each unique feature combination, crafting a personalized clustering model. Following this, we perform binarization based on the identified clusters.

#### 2.3.2. Feature Selection

In this step, we introduce a module to select the personalized feature set. To create the personalized bio-key, the subject who wants to enroll must initiate the bio-key generation step with a distinct feature set. We used the ReliefF algorithm for feature selection to accommodate these distinct individual characteristics [20]. We executed the following process to construct the personalized feature set.

1. Selecting the subject from the experimental DB as the representative example, we extract the feature data (M) and the corresponding number of feature sets (n) (Equation (5)).
  (5) $$M=\{X_{i}|1\leq i\leq n\}$$
  where $X_{i}$ is the feature set that was extracted from *i*th heartbeat.
2. Control group feature data (N) are randomly extracted from the open ECG-ID DB, corresponding to n (Equation (6)).
  (6) $$N=\{X_{i}|1\leq i\leq n\}$$
  Here, x is the feature set extracted from a single heartbeat.
3. Subsequently, the two feature data sets from the previous steps are combined and furnished as input ($Y_{input}$) to the ReliefF algorithm (Equation (7)).
  (7) $$Y_{input}=M\cup N$$
4. Within the ReliefF algorithm ($R\left(\cdot \right))$, the weight assigned to each feature is iteratively updated. This involves identifying the nearest neighbor within the same class and the closest neighbor from the other classes for each data instance.
5. The most distinguishing features for each individual are selected based on these recalibrated weights. The top 10 features ($F_{idx}$) are chosen and designated as the individual’s unique feature set (Equation (8)).
  (8) $$F_{idx}=select(R\left(Y_{input}\right)),$$
6. These top feature indexes for each participant are securely stored within their ID to be subsequently accessed during the registration and authentication phases and employed in reconstructing the encryption key.

#### 2.3.3. Clustering Model

ECG features have subtle fluctuations despite attempts to minimize signal variability through processing. While authentication applications often tolerate these minor changes, cryptographic keys are susceptible to even subtle variations [13]. Therefore, we incorporated three main procedures to generate seeds demonstrating robustness in the face of intra-individual feature variability (Algorithm 1).

Before proceeding with the proposed procedure, a unique step was introduced to classify features according to common trends observed in the population. This step involves applying the 10 selected feature indexes for each individual in the experimental DB to the data in the ECG-ID DB. The result is clustered training data based on individual feature indexes.

Procedure 1 assesses multicollinearity among features to enhance the performance of the clustering model. To facilitate this, we conducted a Pearson correlation analysis on the ECG feature combinations before using them as training data for the model, assessing multicollinearity against established standards. In Procedure 2, we generate a k-means clustering model using data (*I’*) comprised solely of feature combinations that do not exhibit multicollinearity. The ‘*k*’ = [2, 4] is chosen for subsequent binary conversion, with the optimal ‘*k*’ value determined by the silhouette score [21]. Typically, relying solely on the silhouette score may lead to biased clusters. Investigating how biased probabilities can influence the generation of cryptographic keys during key creation is essential. In this study, we analyzed the results based on two criteria for selecting the *‘k’* value from the bio-key perspective: when the silhouette score is at its maximum ($p_{m}$) and when the silhouette score meets the standard with uniformly formed clusters ($p_{u}$). Finally, Procedure 3 involves creating the clustering model with the personalized information gleaned from individuals in Procedures 1 and 2. This information, specifically the clustering model and the feature indexes for each clustering model, is stored as structure type data, $H_{clust}$, for future authentication purposes.

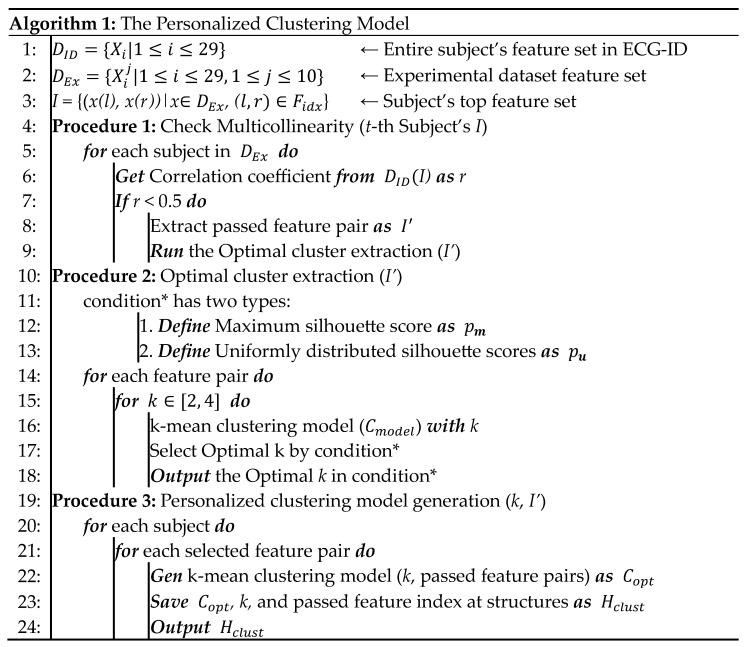



#### 2.3.4. Bit Quantization

In this phase, the classified cluster values are transformed into bit sequences. The feature set is input into the clustering model, each generated based on the top feature index. Subsequently, binarization is conducted for each cluster:

(9) $$B\left(C\right)=bin(C-1,\frac{k}{2})$$

where *C* is the cluster label, *B* is the bit of cluster label, and bin(α,β) is the function that converts α into a binary bit of length β.

The extracted bits are arranged following the sequence of the clustering model, forming a unified bit sequence. This sequence is extended to a length of $2^{n}$ through zero padding to facilitate its input into the fuzzy extractor. The augmented sequence is defined as ‘*seed*’. Concurrently, the count of bits appended is recorded in $H_{clust}$.

### 2.4. Bio-Key Generation

#### 2.4.1. Fuzzy Extractor-Based Key Generation

This section delineates the methodology for generating the bio-key using the proposed seed. The fuzzy extractor was chosen among several algorithms that transform the seed into an encryption key [22]. The output string from this process serves as the personalized bio-key. There are two primary reasons for selecting the fuzzy extractor. First, it is recognized for its efficiency in producing stable encryption keys from sources that exhibit physical variability, a common feature in biometric authentication. Second, it is adept at generating encryption keys with high entropy and unpredictability from various data sources.

The produced seed is fed into the extraction stage of the fuzzy extractor, from which we derive the internally generated helper data ($H_{fz}$) and the encryption key. We refer to the encryption key produced by the fuzzy extractor as the bio-key. The generated $H_{fz}$, in conjunction with $H_{clust}$, is subsequently employed to generate a bio-key from signals measured at different times.

(10) $$[R,H_{fz}]=\mathrm{Gen}(P,l,\;\tau )$$

Here, *Gen* is the key generation function of the fuzzy extractor, *P* is the personalized seed, *l* denotes the length of the key, τ indicates the number of bits that can be flipped in the source value, and *R* is the bio-key. In bio-key generation based on fuzzy extractors, selecting the appropriate τ is crucial. For this aspect, we conduct the heuristic analysis based on authentication performance to determine the optimal τ value.

#### 2.4.2. Enrollment Process

The enrollment process is critical in creating the personal bio-key for subsequent authentication. In this step, we use an example focusing on one subject from the experimental DB. The system inputs feature sets extracted from the subject’s first measurement time $T_{0}$ into the subject-personalized clustering model. The extracted clusters were processed through a voting method to form a single cluster set, and the single cluster set was transformed into the seed. Subsequently, the seed was converted into bio-key and helper data using the ‘*Gen*’ function of the fuzzy extractor. The produced bio-key was stored in the ID along with $H_{fz}$ and $H_{clust}$. Then, the produced bio-key was compared with the reproduced bio-key in the authentication phase.

#### 2.4.3. Authentication Process

The authentication process of individual identity compares the bio-key stored in the ID with that generated from the other ECG. We describe an example using one subject from the experimental DB. The system reproduced the bio-key using the information stored in $H_{fz}$ and $H_{clust}$ within ID alongside data measured at various times $T_{k}$. Unlike the enrollment process, the bio-key was generated from the feature set extracted from a single ensemble beat. The freshly generated bio-key is compared to that stored in the ID.

In this study, our primary focus lies in utilizing electrocardiogram signals for key generation without delving into the specifics of identity storage. Therefore, we assume the generated information and ID are stored securely in a robust cloud system.

#### 2.4.4. Performance Assessment

In this paper, we evaluated the proposed method from three perspectives. First, we compared the performance of our Dynamic Quantization approach with that of existing seed generation studies to assess its effectiveness. Second, to evaluate the randomness of the bio-key and its capability as an encryption key, we applied three tests commonly used for randomness evaluation. Last, we determined the feasibility of using the generated bio-key for personal authentication. We also analyzed the optimal values of the hyper-parameters employed in our method and the conditions outlined in Section 2.3.3 for selecting the optimal cluster.

## 3. Results

This section presents the results of our analysis, which are organized into three distinct aspects for clarity. In alignment with the structure outlined in Section 2.4.4, we evaluate the Dynamic Quantization approach’s effectiveness in Section 3.1. Subsequently, we examine the bio-key randomness and encryption key performance in Section 3.2. Finally, the personal authentication performance of the proposed bio-key is provided in Section 3.3. The analysis was performed using the following laboratory equipment: Intel(R) Core(TM) i7-7700 CPU, 3.60 GHz, 32 GB of RAM, One RTX 3080Ti 11 GB, and MATLAB Toolbox.

### 3.1. Assessment of Dynamic Quantization Effectiveness

#### 3.1.1. Binarization Performance Indicators

We define an ‘error seed’ as a seed discordant with that used in the enrollment. We calculate evaluation indicators, such as error seed rate and error bit rate, to explain the results of Dynamic Quantization. The evaluation indicators are expressed as follows:

(11) $$Error\;Seed\;Rate\;[\%]=\frac{N_{E}^{s}}{N_{T}^{s}}\times 100$$

(12) $$Error\;Bit\;Rate\;[\%]=\frac{N_{E}^{b}}{L}\times 100$$

Here, $N_{E}^{s}$ is the number of error seeds, $N_{E}^{s}$ is the number of total seeds, $N_{E}^{b}$ is the number of bits in the error seed, and *L* denotes the length of the seed. 

#### 3.1.2. Comparative Analysis of Clustering-Based Dynamic Quantization

We implement the binarization method from a previous study to evaluate our proposed method against previous research and compared the seed generation [23]. We analyze performance differences by examining the error seed rate at different *L* values. In contrast to the previous study, which had a fixed *L* of 152 bits, our proposed method had a variable *L* tailored to each individual, spanning from 32 to 57 bits.

When using the clustering method to binarize ECG features, we compared the results with those from the previous study, as shown in Table 2. Excluding the data used for subject registration in the experimental DB, 531 seeds were generated. Our method showed an average of 3.4 error seeds per individual, a promising outcome compared to previous research. Additionally, the average error seed rate was 6.4%, indicating the efficiency of our approach compared to the previous study.

Furthermore, we analyzed the error bit rates by comparing the bits differing from the seed of the enrolled key within the labeled error seeds. Our proposed method differs by an average of 15.04% of the bits of the total seed length within the error seed, a 27.41% improvement in performance compared to prior studies.

### 3.2. Evaluation of Bio-Key Randomness as a Cryptograph Key

#### 3.2.1. Randomness Indicators

In our study, we evaluated the randomness of the generated key from two perspectives: entropy and the pass rate of NIST statistical benchmarks [13,15]. We calculated the Shannon entropy and Min-entropy for the bio-key generated from each subject’s ECG to assess randomness through entropy as in Equations (13) and (14), where $P_{i}$ is the probability mass function, and i∈{0,1}.

(13) $$Min\;Entropy=-{log}_{2}(\underset{i}{\max}\left\{P_{i}\}\right)$$

(14) $$Shannon\;Entropy=-\sum_{i}P_{i}{log}_{2}P_{i}$$

The NIST benchmark, a tool for evaluating the randomness of cryptographic random numbers, was utilized to validate our results further. Four main tests from the NIST suite were implemented in this study: the Frequency Monobit Test (F-test), the Frequency Test within a Block (B-test), the Runs Test (R-test), and the Longest Run of Ones in a Block (L-test).

#### 3.2.2. Results of Randomness for a Bio-Key

Table 3 presents the performance metrics of our proposed method in terms of entropy, showing a minimum entropy of 0.77 and a Shannon entropy of 0.96. These figures surpass the results of prior studies by 0.23 and 0.13, respectively, indicating that the seeds generated by our method exhibit greater irregularity and unpredictability. Moreover, our approach has the distinction of successfully passing all NIST benchmarks, underscoring its robustness. In addition to these metrics, our method’s pass rate (also called *p*-value) exceeds that of previous studies in most tests, demonstrating its superior performance in ensuring the randomness and security of generated seeds.

The proposed method yields a *p*-value of 0.50 in the R-test, marginally lower than the 0.63 recorded for the previous method. It is crucial to contextualize this result within the broader scope of the performance metrics. The R-test is just one of the indicators employed to ascertain the randomness of our method. A holistic performance assessment should incorporate all metrics and not focus on a single test. Our method results across various tests, including the F-test and L-test, demonstrate the balanced and secure results of bio-key authentication.

### 3.3. Personal Authentication Performance of the Bio-Key

We reproduced a total of 531 bio-keys for 10 subjects, encompassing data measured at different times for each subject. We obtained 531 reproduced bio-keys for each individual using the helper data in one individual’s ID during the authentication process. Then, we extracted authentication performance based on the bio-key registered in the ID.

#### 3.3.1. Authentication Performance Indicators

We employ three commonly used biometric indicators and a template-matching method to assess the authentication performance of the generated bio-key. In particular, striking a balance among the three indicators is crucial in the authentication domain, and finding the optimal balance among them is important. The evaluation indicators are defined as follows:

(15) $$Accuracy=\frac{TP+TN}{TP+TN+FP+FN}$$

(16) $$False\;Accept\;Rate(FAR)=\frac{FP}{FP+FN}$$

(17) $$False\;Reject\;Rate(FAR)=\frac{FN}{TP+FN}$$

Here, TP is the number of true-positives, FP is false-positives, FN is the number of false-negatives, and TN is the number of true-negatives.

#### 3.3.2. Bio-Key-Based Authentication Results

Table 4 shows the authentication performance achieved by comparing bio-keys re-generated from ECG data measured at different times with the enrolled bio-keys. Three parameters were considered for authentication performance assessment: the clustering parameter (*k*), the criterion for selecting the optimal number of clusters ($p_{m}$ or $p_{u}$), and the fuzzy extractor parameter (τ). Through these evaluations, we confirmed that the parameter combination yielding the best performance delivers 95% accuracy, 5% FAR, and 2% FRR at *k* = dynamic allocation, with $p_{u}$ and τ = 0.05. Each optimal parameter value was selected for the following reasons:

1. For *k* = 2, *k* = 4, and *k* = [2, 4], the average accuracies were 72%, 89%, and 88%, respectively. Among these, *k* = [2, 4] exhibited a balanced performance in FAR and FRR compared to other *k* values and is optimal.
2. With *k* = [2, 4] fixed, evaluation under optimal cluster selection conditions revealed that $p_{u}$ outperformed $p_{m}$ by an average of 11% in accuracy, 14% in FAR, and 2% in FRR, indicating that $p_{u}$ is optimal.
3. As τ increases, FRR performance improves, but ACC performance decreases. Considering the balance across all three indicators, τ = 0.05 is optimal.

Detailed explanations for each reason will be provided in the following paragraphs.

**Figure 4:**
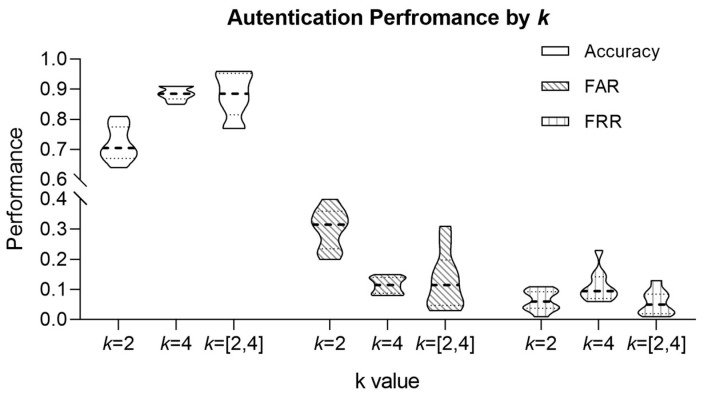
Performance comparison results according to clustering model parameter *k*.

Figure 4 delineates the bio-key authentication performance metrics as a function of the number of clusters (‘*k*’). In constructing the clustering model, we evaluated the performance under fixed and dynamic ‘*k*’ values. Specifically, for the fixed ‘*k*’ of two, we observed an average accuracy of 72%, an average false acceptance rate (FAR) of 30%, and an average false rejection rate (FRR) of 6%. When ‘*k*’ was fixed to four, the average accuracy was 89%, with the corresponding average FAR of 11% and an average FRR of 11%.

Upon evaluating performance with ‘*k*’ = [2, 4], we earned an average accuracy of 88%, an average FAR of 13%, and an average FRR of 6%. Notably, there was no marked difference in average accuracy and FAR for ‘*k*’ = 4 and ‘*k*’ = [2, 4], but a substantial divergence of greater than 5% in FRR was noted. The similarity in performance metrics for average accuracy and FAR was calculated without accounting for cluster selection conditions.

When considering optimal cluster selection conditions, the data show with ‘*k*’ at 4, an average accuracy of 88%, average FAR of 12%, and average FRR of 13%. In contrast, with ‘*k*’ = [2, 4], the average accuracy improves to 94%, the average FAR decreases to 6%, and the average FRR decreases to 5%. These results emphasize the influence of cluster selection conditions on the performance of bio-key authentication and underscore the significance of choosing an appropriate ‘*k*’ value to optimize authentication accuracy and error rates. Moreover, as indicated by the findings in Figure 4, the authentication performance achieved an optimal balance within k = [2, 4].

**Figure 5:**
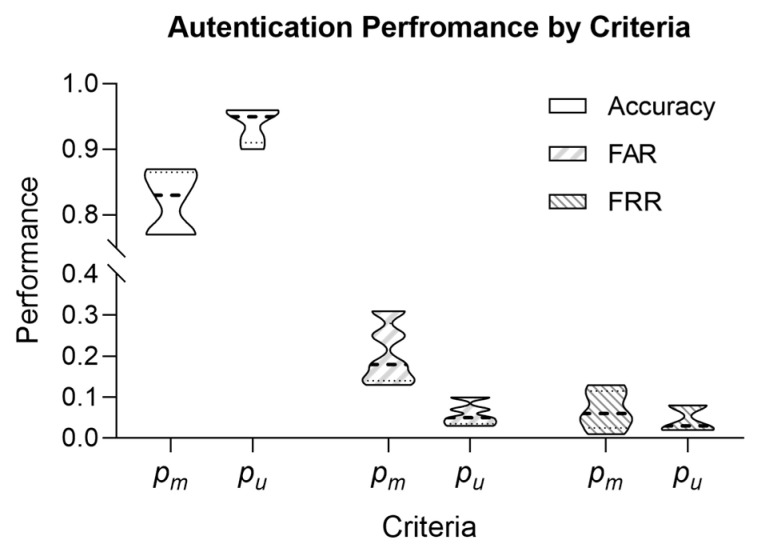
Performance comparison results according to optimal cluster selection conditions.

Figure 5 elucidates the authentication performance of the bio-key contingent on the conditions for cluster selection. As delineated in Section 2.3.3, we applied two criteria to the clustering model for optimal cluster selection.

In conditions $p_{u}$, the authentication performance obtained an accuracy between 90% and 97%, with an FAR between 3% and 10% and an FRR from 2% to 8%. Conversely, in the conditions $p_{m}$, the authentication performance manifested an accuracy between 77% and 87%, with an FAR between 13% and 31% and an FRR from 1% to 13%. These findings suggest that the best conditions for cluster selection, which help to generate and authenticate a bio-key, are achieved when the silhouette score is satisfied and the clusters are evenly distributed. These two cluster selection criteria were evaluated while keeping the remaining parameters at τ = 0.01~0.09 and *k* = [2, 4].

**Figure 6:**
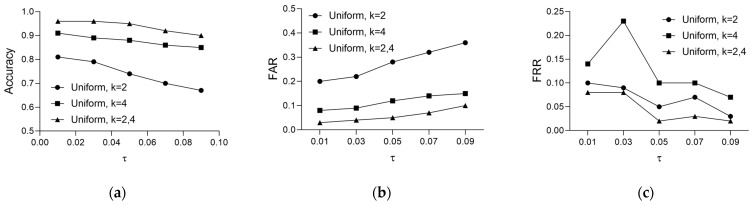
Authentication performance results according to changes in fuzzy extractor parameter τ: (**a**) accuracy, (**b**) FAR, and (**c**) FRR.

Figure 6 illustrates the authentication performance of the bio-key with various fuzzy extractor settings. Our method achieved a maximum of 96% accuracy, with a minimum of 3% FAR and 2% FRR, depending on the parameter ‘τ.’

Figure 6 shows that, as ‘τ’ increases from 0.01 to 0.09, the accuracy decreases by up to 3% and by at least a minimum of 1%. In comparison, the FRR decreases by a maximum of 6% and a minimum of 1%. This result indicates an inverse proportional relationship. Conversely, the FAR shows a proportional relationship, increasing by a maximum of 3% and a minimum of 1%. These findings suggest that, as ‘τ’ increases, the system becomes more user-friendly for registrants, but security is compromised due to the higher likelihood of false acceptance of non-registrants.

## 4. Discussion

The ECG-based bio-key generation method faces hurdles due to the variability of the signal, making it challenging to regenerate the same encryption key from a later signal [24,25]. In this study, we propose a novel approach called Dynamic Quantization that considers the variability of ECG signals in generating stable seeds. Our proposed clustering-based binarization method is compared to previous research regarding error seed rate and error bit rate (Table 2). Our method classifies only 6.4% of all seeds as error seeds, even when dealing with signals measured at different times from the same individual. In contrast, previous research did not incorporate techniques to mitigate the variability of ECG signals, resulting in a mismatch among seeds. The proposed method minimizes the variability of ECG signals caused by different external conditions and individual states to generate stable seeds even at different time points.

Figure 4 compares authentication performance between fixed *k* and varying *k* when *k* = [2, 4]. The results demonstrate that, when *k* varies according to feature combinations, the average accuracy, FAR, and FRR significantly improve to 94%, 6%, and 5%, respectively. These findings affirm the superior performance achieved by adapting *k* based on feature combinations. From these results, our proposed method generates encryption keys of varying lengths for each individual, spanning from 32 to 57 bits. The proposed method offers a relatively short key length of 32 to 57 bits compared to traditional encryption techniques like AES-128. Such shorter keys are suitable for applications requiring low capacity and processing power, such as PDAs, wireless networks, and embedded devices [26]. Also, the proposed keys can be used as pre-shared keys for systems requiring longer encryption keys by leveraging key derivation functions like SHA-2 hash or PBKDF2 [27,28].

The importance of this paper is that the proposed method offers several advantages, particularly in enhancing the security and flexibility of the encryption system. Previous studies have explored strategies like double security with a single key length and the generation of long-length keys to bolster security [29,30]. The existing methods may not be viable in resource-constrained environments [31]. In contrast, the unique feature of our proposed method, which generates keys of different lengths for each individual, enhances security by employing a complex feature combination structure in limited scenarios and optimizes resource allocation. In these ways, it improves system efficiency in situations with constrained computational capabilities, ensuring enhanced security.

In this study, we introduced a framework that generates bio-keys using ECG signals within a notably brief measurement period. Conventionally, ECG-based encryption keys predominantly utilize the IPI feature of the ECG signal. This method typically requires identifying two successive R peak points in the ECG, necessitating a continuous measurement duration ranging from 30 to 300 s for generating a viable encryption key [5,6]. Furthermore, approaches employing non-fiducial ECG features also demand measurement times of a minimum of 180 s [13,32]. However, these existing methodologies pose practical limitations due to their extended measurement durations required for authentication [33]. In contrast, our proposed method significantly shortens this process. Our method can generate a bio-key from each ensemble heartbeat, requiring only 20 s for initial registration and a minimum of 5 s for subsequent authentication. Reducing ECG measurement time enhances user convenience and is crucial in applications where rapid authentication is essential.

Previous studies have often utilized large-scale databases, with ECG measurements obtained in hospital settings using wet electrodes and specialized equipment [6,13,32]. In contrast, our study involved a smaller participant pool [5,11,12]. However, the key distinction of our approach is dry electrodes, which are more common in wearable devices, for ECG measurements at varying times [34,35]. This aspect of our research is particularly relevant for practical applications, as it reflects the varying electrode positions over time. Additionally, our study is a preliminary investigation into the feasibility of generating time-invariant bio-keys from ECG signals. Our findings demonstrate promising results, achieving up to 0.99 entropy. Furthermore, we observed an accuracy of 96%, an FAR of 5%, and an FRR of 6% for bio-keys measured at different times. These outcomes provide a solid framework for advancing to subsequent phases of research.

## 5. Conclusions

Bio-key generation should accommodate individual ECG variability and be flexible and user-friendly. This paper introduces a robust method for generating bio-keys that exhibit excellent randomness and stability. Furthermore, we consider adding flexible, personalized seed generation capabilities for bio-key generation. We tested our method using data collected from dry electrodes at various times. Our approach demonstrates a maximum entropy of 0.99 and an authentication accuracy of 96%, showing its resilience to individual ECG variations. These research findings pave the way for practical service offerings across various security systems.

In future research, evaluating authentication performance using a larger sample size will be crucial. Additionally, assessing ECG variations within and across different sessions, considering various physiological states, is essential. We intend to collect data under diverse physiological conditions and from commercial products to overcome this limitation. In doing so, our results will provide valuable insights for future research directions.

## Figures and Tables

**Table 1:** Feature set configuration table extracted from a single ensemble heartbeat.

Category	Features					
Amplitude	1	ST amplitude	2	RS amplitude	3	RQ amplitude
	4	PQ amplitude	5	PR amplitude	6	RT amplitude
	7	PS amplitude	8	PT amplitude	9	QT amplitude
Duration	10	QS interval	11	QR interval	12	RS interval
	13	PR interval	14	RT interval	15	ST interval
	16	PQ interval	17	PT interval	18	QT interval
Slope, Distance, and Area	19	PR slop	20	PQ slop	21	QS slop
	22	ST slop	23	RT slop	24	PR distance
	25	PQ distance	26	QS distance	27	ST distance
	28	RT distance	29	QRS area		

**Table 2:** Table of error seeds and error bit numbers and ratios by method.

Method	Total Seeds	Error Seeds	Error Seed Rate	Error Bits	Error Bit Rate
	[N]	[N]	[%]	[N]	[%]
Camara et al. [23]	531	531	100	64.53	42.45
(*L* = 152 bits)					
Proposed Method	531	34	6.4	6.03	15.04
(*L = n*-bits)					

**Table 3:** Entropy and NIST test results of seeds generated by method and bio-key.

Method	Min Entropy	Shannon Entropy	F-Test	B-Test	R-Test	L-Test
	[-]	[-]	0 Bit/1 Bit [%]	[*p* Value]	[*p* Value]	[*p* Value]
Camara et al. [23]	0.53	0.89	41.95/58.05	0.41	0.63	0.58
Proposed Method	0.77	0.96	44.77/55.23	0.53	0.50	0.63
Proposed Method + Fuzzy Extractor	0.88	0.99	51.88/48.12	0.56	0.70	0.63

**Table 4:** Authentication performance of the proposed method under various conditions.

		Accuracy					FAR					FRR				
		τ (Hamming Error Bit Ratio)														
Criteria	*k*	0.01	0.03	0.05	0.07	0.09	0.01	0.03	0.05	0.07	0.09	0.01	0.03	0.05	0.07	0.09
$p_{m}$ (Max Silhouette Score)	2	0.77	0.71	0.69	0.67	0.64	0.24	0.31	0.33	0.36	0.4	0.11	0.09	0.04	0.04	0.01
	4	0.91	0.91	0.89	0.88	0.87	0.08	0.1	0.11	0.13	0.14	0.15	0.09	0.09	0.07	0.06
	[2, 4]	0.87	0.86	0.83	0.77	0.77	0.13	0.15	0.18	0.25	0.31	0.13	0.1	0.06	0.04	0.01
$p_{u}$ (Uniformity Silhouette Score)	2	0.81	0.79	0.74	0.7	0.67	0.2	0.22	0.28	0.32	0.36	0.1	0.09	0.05	0.07	0.03
	4	0.91	0.89	0.88	0.86	0.85	0.08	0.09	0.12	0.14	0.15	0.14	0.23	0.1	0.1	0.07
	[2, 4]	0.96	0.96	**0.95**	0.92	0.9	0.03	0.04	**0.05**	0.07	0.1	0.08	0.08	**0.02**	0.03	0.02

The bolded results indicate the outcome of the optimal parameter combination.

## Data Availability

Data are contained within the article.
